# Supplementary material for: The Combination of GS-441524 (Remdesivir) and Ribavirin Results in a Potent Antiviral Effect Against Human Parainfluenza Virus 3 Infection in Human Airway Epithelial Cell Cultures and in a Mouse Infection Model
Source: Viruses. 2025 Jan 26;17(2):172. doi: 10.3390/v17020172 (PMC11860817; doi:10.3390/v17020172)
Supplement: Supplementary file 1 [file viruses-17-00172-s001.zip › viruses-3440545-supplementary.pdf]

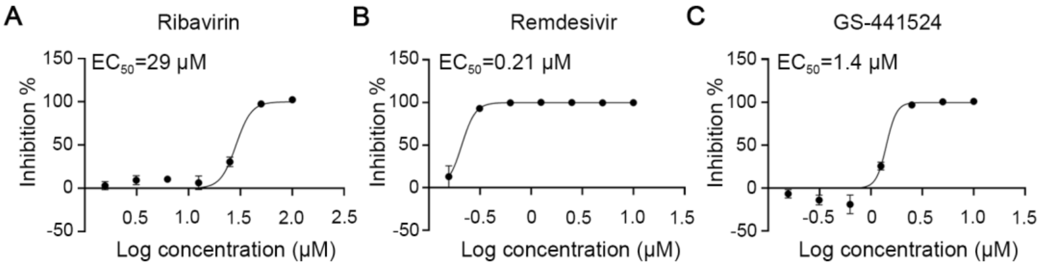

D

Ribavirin/ μM	25	30.9	32.1	32.9	33.4	32.5	33.0	35.4
	12.5	22.7	23.1	23.6	24.1	24.2	26.4	28.2
	6.25	11.2	12.6	13.1	13.8	13.4	11.3	7.6
	3.13	7.3	7.6	10.2	10.4	9.5	5.4	0.0
	1.56	-0.3	6.9	6.5	6.2	5.0	0.3	-4.6
	0	0.0	3.5	2.7	3.8	1.4	-0.9	-8.8
	**Toxicity%**	0	0.03	0.06	0.13	0.25	0.5	1
		**Remdesivir/μM**						

E

Ribavirin/ μM	25	24.3	26.1	26.9	28.4	25.9	24.1	24.3
	15	22.3	23.9	23.5	24.0	25.0	21.3	20.4
	10	18.8	19.3	20.6	18.5	18.7	17.9	15.7
	5	10.4	8.2	16.3	15.4	12.8	11.1	10.6
	2.5	4.0	8.1	8.7	11.8	11.6	9.6	4.7
	0	0.0	2.3	3.8	4.3	5.0	2.8	1.0
	**Toxicity%**	0.00	0.04	0.12	0.37	1.11	3.33	10
		**GS-441524/μM**						

Figure S1. (A-C) Percentage inhibition of HPIV-3 replication, measured as GFP-signal, normalized to the untreated-infected control. **(D, E)** The matrices of toxicity in the presence of combined treatments. Data are from three independent experiments.

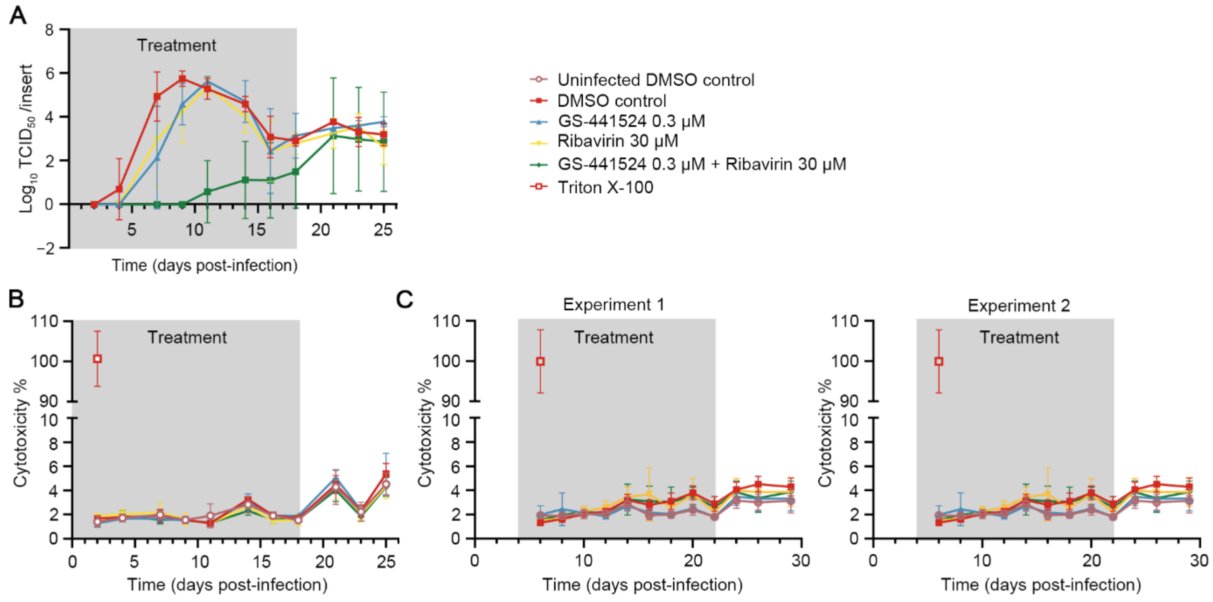

Figure S2. (A) Infectious virus titers in apical washes from Experiment 2 were determined by endpoint virus titration assay in the prophylactic setting. The lower limit of detection is -0.02. **(B, C)** Neither ribavirin nor GS-441524 has obvious cytotoxicity in HnAEC cultures in both the prophylactic setting **(B)** and the therapeutic setting **(C)**. Data presented for Experiment 1 and Experiment 2 are two independent experiments. The grey boxes indicate treatment windows.

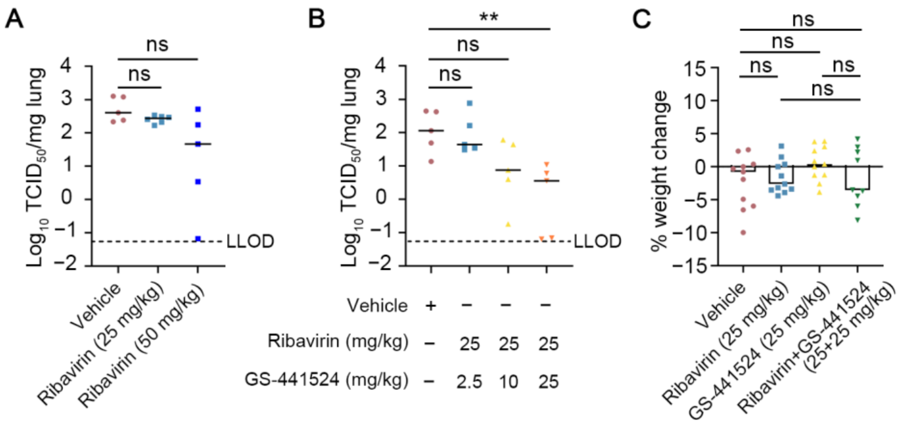

Figure S3. Effect of ribavirin and GS-441524 against HPIV-3 replication in AG129 mice. (A) AG129 mice were orally treated with ribavirin (either 25 mg/kg or 50 mg/kg) or the vehicle (i.e. the control group) twice daily (BID) for 4 consecutive days, starting one day before intranasal inoculation with HPIV-3. At day 3 p.i., mice were euthanized and lung samples were collected to quantify infectious virus titers. Data are from one experiment with 5 or 6 mice per group. **(B)** AG129 mice were orally treated with the vehicle and the combinations of ribavirin and various doses of GS-441524 (2.5 mg/kg, 10 mg/kg and 25 mg/kg) twice daily for 4 consecutive days, starting one day before intranasal inoculation. At day 3 p.i., lung samples were collected to quantify infectious virus titers. Data are from one experiment with 5 mice per group. **(C)** Weight change at day 6 p.i. is presented as a percentage of the body weight at day −1 p.i., when treatment was started. Data are from two independent experiments with 9 mice in the combination-treated group and 11 mice in all other groups. LLOD presents the lower limit of detection. Individual data and median values (indicated by bars) are presented in all graphs. Data were analyzed with the Kruskal-Wallis test. ns, nonsignificant; $p<0.01$, **.
